# Properties of Calcium Phosphate Cements With Different Tetracalcium Phosphate and Dicalcium Phosphate Anhydrous Molar Ratios

**DOI:** 10.6028/jres.113.025

**Published:** 2008-12-01

**Authors:** Satoshi Hirayama, Shozo Takagi, Milenko Markovic, Laurence C. Chow

**Affiliations:** National Institute of Standards and Technology, Gaithersburg, MD 20899-0001

**Keywords:** absorption, calcium phosphate, calcium phosphate cements, hydroxyapatite, mechanical properties, porosity

## Abstract

Calcium phosphate cements (CPCs) were prepared using mixtures of tetracalcium phosphate (TTCP) and dicalcium phosphate anhydrous (DCPA), with TTCP/DCPA molar ratios of 1/1, 1/2, or 1/3, with the powder and water as the liquid. Diametral tensile strength (DTS), porosity, and phase composition (powder x-ray diffraction) were determined after the set specimens have been immersed in a physiological-like solution (PLS) for 1 d, 5 d, and 10 d. Cement dissolution rates in an acidified PLS were measured using a dual constant composition method. Setting times ((30 ± 1) min) were the same for all cements. DTS decreased with decreasing TTCP/DCPA ratio and, in some cases, also decreased with PLS immersion time. Porosity and hydroxyapatite (HA) formation increased with PLS immersion time. Cements with TTCP/DCPA ratios of 1/2 and 1/3, which formed calcium-deficient HA, dissolved more rapidly than the cement with a ratio of 1/1. In conclusion, cements may be prepared with a range of TTCP/DCPA ratios, and those with lower ratio had lower strengths but dissolved more rapidly in acidified PLS.

## 1. Introduction

Water-activated self-hardening calcium phosphate cements (CPCs) have been the subject of considerable interest in biomaterial research, especially as bone graft materials. A CPC that consisted of equimolar amounts of tetracalcium phosphate (TTCP), Ca_4_(PO_4_)_2_O, and dicalcium phosphate anhydrous (DCPA), CaHPO_4_, was the first such material in clinical use. It was shown to harden in about 30 min when water was used as the cement liquid and formed hydroxyapatite (HA) as the product. A previous study showed that the TTCP/DCPA cements can harden satisfactorily and develop reasonable strengths when the TTCP/DCPA molar ratio was as low as ½ [1]. Another study [2] investigated properties of cements with TTCP/DCPA ratios ranging from 0.25 to 2.0. This study reported the pH of cement slurries during setting, and the strengths and HA conversion of 1-d cement samples.

Calcium-deficient HA with a Ca/P molar ratio lower than 1.67, the ratio for stoichiometric HA, were formed in cements with a TTCP/DCPA ratio less than 1.0 [2]. Calcium-deficient HA is reported to be more soluble and therefore may resorb more rapidly *in vivo* than stoichiometric HA [3]. Dissolution rate measurements under constant-composition conditions were previously proposed [4] as an *in vitro* model for estimating relative in vivo resorption rates for calcium phosphate bone graft materials. The present study investigated the dissolution rate of 1-d cement samples with TTCP/DCPA molar ratios of 1/1, 1/2, and 1/3, yielding overall Ca/P molar ratios of 1.67, 1.50, and 1.33, respectively. Extent of HA conversion, diametral tensile strength (DTS), and porosity of cement samples of varying TTCP/DCPA ratios that had been immersed in a physiological-like solution (PLS) for 1 d, 5 d, and 10 d were also determined.

## 2. Materials and Methods

### 2.1 Preparation of Calcium Phosphate Cements (CPCs)

TTCP was prepared by heating an equimolar mixture of commercially obtained DCPA and CaCO_3_ (J. T. Baker Chemical Co., NJ, USA)^1^ at 1500 °C for 6 h in a furnace (Model 51333, Lindberg, Watertown, WI) and quenched at room temperature. TTCP and commercially obtained DCPA were ground in a planetary ball mill (Retsch PM 4, Brinkman, NY) to obtain the desired medium particle sizes of 17 μm and 1 μm, respectively. The particle sizes of TTCP and DCPA were measured using a centrifugal particle size analyzer (SA-CP3, Shimadzu, Kyoto, Japan).

Three CPC powders were prepared by homogeneously mixing appropriate amounts of the ground TTCP and DCPA to produce materials with TTCP/DCPA molar ratios of 1:1 (CPC(1/1)), 1:2 (CPC(1/2)) and 1:3 (CPC(1/3)).

### 2.2 Setting Time Measurement

Distilled water was used as the cement liquid. To produce pastes of similar consistencies, powder to liquid (P/L) mass ratios of 4.0, 3.5 and 3.3 were used for CPC(1/1), CPC(1/2) and CPC(1/3), respectively. The setting time measurements were performed on 3 specimens for each the three CPC formulations using the Gillmore needle method as follows. CPC powder (0.3 g) and distilled water were mixed at the designated powder/liquid (P/L) ratio to produce a smooth paste which was packed into a stainless steel mold (6.0 mm diameter (D) × 3.0 mm high (H)). Both ends of the mold were then covered with glass plates held by a C-clamp, and the mold was then placed in a 100 % relative humidity box at 37 °C. Periodically, the specimen was removed from the box for the setting time test. The specimen was considered set when the Gillmore needle, with 453 g of mass loaded on a needle with a tip diameter of 1.0 mm, failed to make a perceptible circular indentation on the surface of the specimen [5]. The mean setting time value (*n* = 3) and standard deviation (denoted by the ± symbol) are reported. For setting time and other measured quantities described in this paper, the standard deviation is used as a measure of the standard uncertainty.

### 2.3 DTS Sample Preparation

A previously reported method [6] for preparing specimens for diametral tensile strength (DTS) measurements was used. Briefly, the mold was a stainless steel rod (25.4 mm D × 30 mm H) with a 6 mm diameter hole drilled through the center and the top 6 mm length of the cylinder machined to a funnel shape. To prepare a specimen, the mold body was first placed in a sleeve, and a stainless steel plunger (6 mm D × 20 mm H) was dropped into the mold cavity, allowing 6 mm of the plunger to protrude beyond the bottom surface of the mold. A weighed amount of CPC powder (0.18 g) and the appropriate amount of distilled water were mixed on a glass mixing slab with a stainless steel spatula. After a mixing time of 30 s, the cement paste was packed into the mold cavity, and the top plunger of the same dimensions was placed into the mold cavity. Approximately 7 mm of the plunger protruded above the top surface of the mold. The mold assembly was then placed in the constant pressure-loading device, and a pressure of 0.7 MPa was applied to the cement specimen through the top and bottom plungers for 4 h at 37 °C in a 100 % relative humidity box. The hardened specimen was then removed from the mold and stored in 30 mL of a physiological-like solution (PLS) for 1 d, 5 d, or 10 d at 37 °C with the solution being replaced daily. PLS has a composition ([CaCl_2_] = 1.15 mmol/L, [KH_2_PO_4_] = 1.20 mmol/L, [NaCl] = 133 mmol/L, [HEPES] = 50 mmol/L, and pH = 7.4) similar to that of serum in terms of calcium, phosphate and electrolyte contents [7]. This method was also used for preparing samples for the porosity and dissolution rate measurements described below.

### 2.4 DTS Measurement

DTS measurements were conducted using a Universal Testing Machine (Instron, Model 5500, United Calibration Co., Garden Grove, CA). The diameter and height of each specimen were first measured with a micrometer. The specimen was then placed between steel platens each covered with a layer of wet filter paper and crushed at a displacement rate of 10 mm/min. The DTS value was the average of values obtained from 5 specimens.

### 2.5 Measurement of HA Formation

The conversion of CPC cement components to HA was calculated from powder x-ray diffraction (XRD) peak intensities. After the DTS measurement, the specimen was dried and ground to fine powders and characterized by XRD. The XRD patterns were recorded (Rigaku DMAX 2200, Danvers, MA, U.S.A.) using graphite-monochromatized CuK*_α_* radiation (*λ* = 0.154 nm) generated at 40 kV and 25 mA. The specimen was scanned from 20 to 40 degrees 2θ in a continuous mode (2° 2θ min^−1^, time constant 2 s) and peak intensities were recorded on a strip-chart recorder. The relative peak intensities of the 29.2° and 29.8° (two theta) reflections for TTCP, the 26.6° reflection for DCPA, and the 25.9° reflection for HA were recorded a minimum of three times, and the averaged values were used to estimate the amount of CPC conversion to HA as described previously [8]. Values obtained from 3 specimens were averaged to produce the mean HA conversion (%).

### 2.6 Porosity Measurement

The specimens for porosity measurements were prepared using the same method that was previously described for preparing the DTS specimen. After the designated immersion time in PLS, each specimen was dried in an oven at 60 °C for 3 h, cooled in a desiccator for 2 h, and then measured for specimen height (H), diameter (D) and mass (wt). The sample bulk density (d) was calculated from the equation, d = wt / π (D/2)^2^ H, and sample porosity was calculated from the equation, porosity (%) = 100 (1 − d/d_HA_), where d_HA_ (= 3.14 g/cm^3^) is the crystal density of HA [9]. The reported porosity value was the average of values obtained from 5 specimens.

### 2.7 Dissolution Rate Measurement

As an initial assessment of in vivo resorption rates, dissolution rates of CPC specimens with different Ca/P ratios were measured. A solution with ionic composition similar to that of serum ([Ca] = 1.15 mmol/L; [P] = 1.2 mmol/L; [KCl] = 133 mmol/L) [6] except for the pH, which was adjusted to 3.0 with HCl, was used as the demineralizing solution[4]. Because a CPC specimen may not have a homogeneous composition due to unreacted cement components, it is possible that the dissolution rate of a given CPC specimen may change with the amount of dissolution that had occurred. Thus, for CPC of a given Ca/P ratio, the following two types of specimens were used in the dissolution experiments: (1) 1-d samples (see Sec. 2.3), and (2) 50 % predissolved samples—these are 1-d CPC samples that had been immersed in a large volume of the demineralizing solution until approximately half of the sample mass was lost.

Dissolution rate measurements of CPC specimens were conducted following a previously described procedure[4]. Briefly, the measurement was conducted in a jacketed, 100 mL capacity glass vessel connected to a circulating bath set at 37 ºC. A pencil-size combination pH electrode and a calcium ion selective electrode were used as the sensors for triggering delivery of the P- and Ca-titrant solutions, respectively, by two independent automatic titrators (Dosimat 665, Brinkmann Instruments, Westbury, NY). The demineralizing solution (40 mL) was placed in the vessel, and stable pH and Ca electrode readings were obtained under constant stirring (400 rpm). These readings were used as set point for the respective titrators. The CPC specimen with known surface area, attached to a Plexiglas^®^ rod, was then placed in the demineralizing solution. Dissolution of the sample would cause increases in the calcium and phosphate concentrations and the pH, triggering addition of the titrants which, in turn, maintained the demineralizing solution composition essentially constant. A small suction tube connected to a peristaltic pump (Masterflex, Cole-Palmer Instrument Company, Chicago, IL) was placed in the vessel to remove liquid above a preset level of 60 mL so that the volume of the solution was kept constant throughout the rest of the dissolution process.

The dissolution rates were expressed in terms of the amounts of calcium and phosphate dissolved per square centimeter of sample surface area per min (mmol cm^−2^ min^−1^). These rates, which will be referred to as the Ca and P dissolutions rates, were calculated from the rates of Ca-titrant and P-titrant additions, respectively. The rates were then averaged over an approximately 4 h period during which the dissolution proceeded under a steady state condition. The Ca/P ratio of the material undergoing dissolution during this time period, hereafter referred to as Ca/P ratio of dissolution, was calculated from the Ca and P dissolution rates. The dissolution rate was also expressed in terms of μg of HA dissolved per square centimeter of sample surface area per min. This value was calculated from the Ca and P dissolution rates and the formula mass of the dissolving mineral, assuming that the formula of the dissolving mineral is Ca_5 − x_ H_2x_ (PO_4_)_3_OH, where (5 − x)/3 is the Ca/P ratio of dissolution measured during the time period. The mean and standard deviation of dissolution rates measured on 5 specimens are reported.

### 2.8 Statistical Data Analyses

A commercially available statistical analysis software, Kwikstat, (TexasSoft, Cedar Hill, TX) was used to perform one-way or two-way ANOVA (Analysis of Variance) on the data. In the cases where a significant (p < 0.05) difference was detected, Newman-Keuls multiple comparison tests [10] were conducted. In this paper the standard deviations are used as measures of the standard uncertainty.

## 3. Result

### 3.1 Setting Time and Porosity

The setting times of CPC with TTCP/DCPA of 1/1, 1/2 and 1/3 were (mean standard deviation) (30 ± 3) min, (30 ± 1) min and (29 ± 2) min, respectively (Table 1). The setting times were not significantly different for the three groups. The porosity of CPC ranged from 33.3 % to 41.4 % (Table 1a). Two-way ANOVA of the porosity data with CPC group (TTCP/DCPA ratio) and immersion time as independent variables showed (Table 1b) that the porosity was significantly affected by both the immersion time (p = 0.05) and CPC group (p < 0.01). Also, there was a significant (p = 0.014) interaction between the two main effects. As a result, for each immersion time, the porosities of the three CPC groups were always significantly different. In contrast, while CPC(1/1) samples exhibited no differences in porosity among the samples in the three immersion times, samples in the other two CPC groups showed increased porosity with immersion time.

### 3.2 Diametral Tensile Strength

The DTS values ranged from 7.23 MPa to 11.71 MPa (Table 2a). Two-way ANOVA of the DTS data with CPC group (TTCP/DCPA ratio) and immersion time as independent variables showed that DTS was significant (p < 0.01) affected by both the CPC group and the immersion time. There was also a significant (p < 0.01) interaction between the two main effects. For both the 1-d and 5-d immersion times, the DTS for all three CPC groups was significantly different, with DTS decreasing with decreasing TTCP/DCPA ratio, which led to increasing porosity (Table 1a). For the 10-d immersion time, the DTS of CPC(1/1) and CPC(1/2) were not different and both were higher than that of CPC(1/3). Immersion time caused the DTS of CPC(1/1) to decrease while it showed no effects on CPC(1/3). For CPC(1/2), the DTS of the 5-d immersion time was higher than those of the 1-d and 10-d immersion time groups.

### 3.3 XRD Analysis and Degree of HA Conversion

**Fig. 1:**
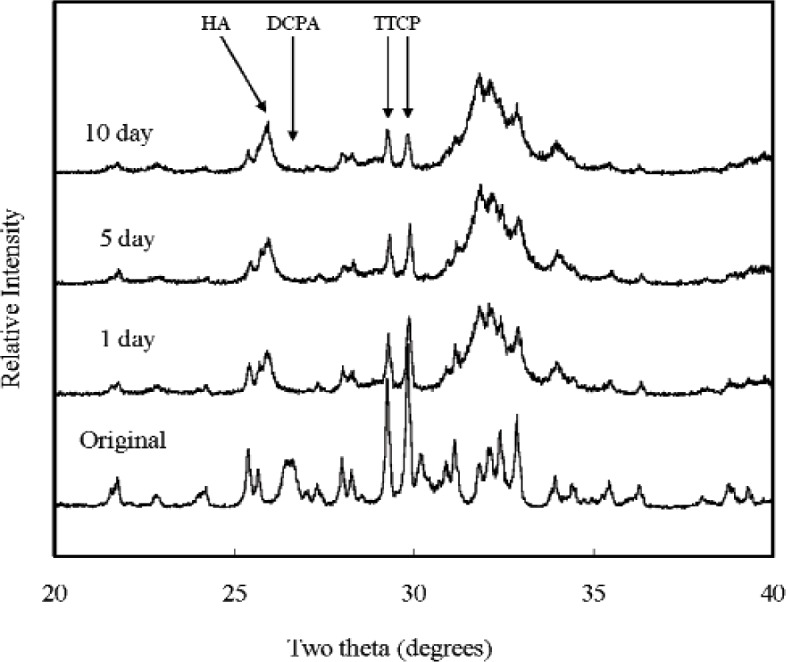
Powder x-ray diffraction patterns of CPC specimens with TTCP/DCPA (1/1) after immersion in the PLS for 1 d, 5 d, and 10 d. The peaks used for the calculation of the HA formation are marked.

**Fig. 2:**
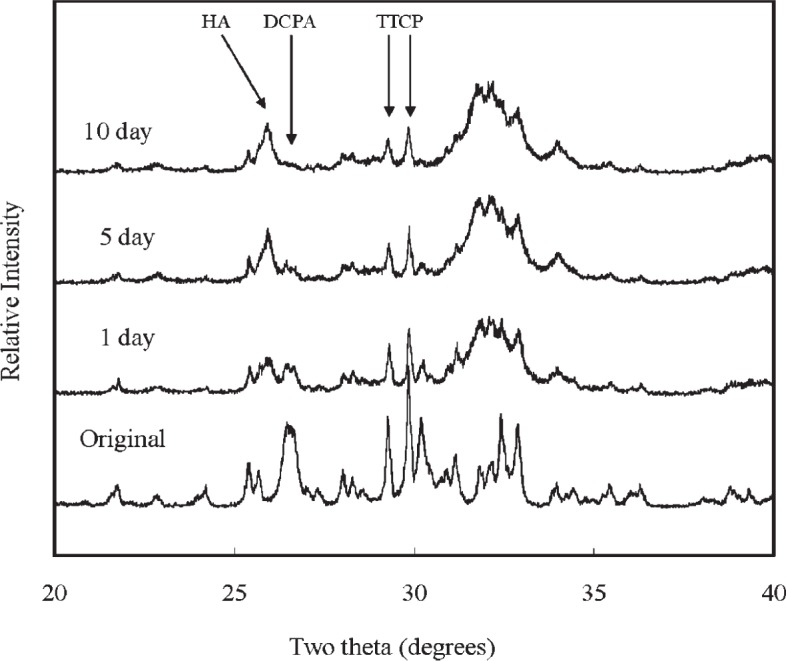
Powder x-ray diffraction patterns of CPC specimens with TTCP/DCPA (1/2) after immersion in the PLS for 1 d, 5 d, and 10 d. The peaks used for the calculation of the HA formation are marked.

**Fig. 3:**
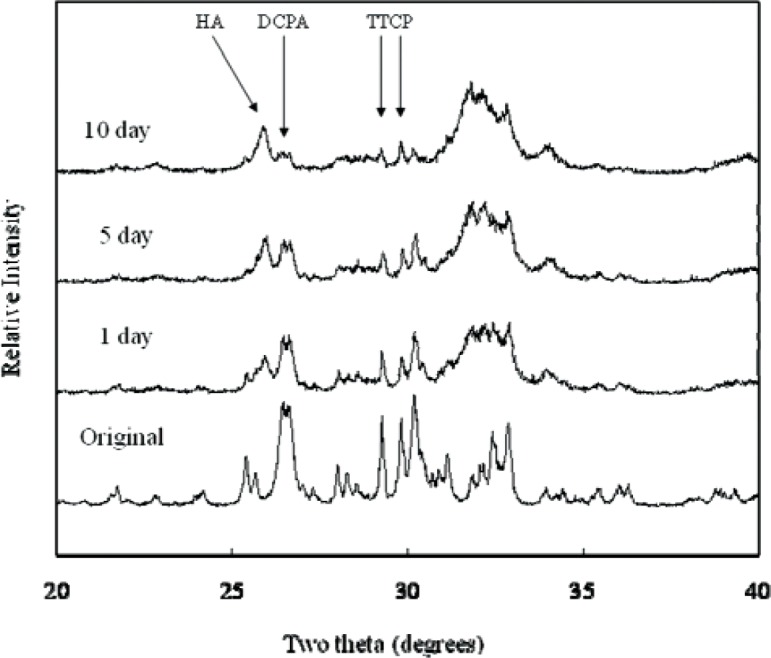
Powder x-ray diffraction patterns of CPC specimens with TTCP/DCPA (1/3) after immersion in the PLS for 1 d, 5 d, and 10 d. The peaks used for the calculation of the HA formation are marked.

XRD analysis revealed that all CPCs were essentially converted to HA at 1 d, with some residual TTCP peaks in CPC(1/1) and residual TTCP and DCPA peaks in CPC (1/2) and CPC (1/3) present. Amounts of TTCP left in the CPC (1/1) decreased during the PLS immersion (Fig. 1). DCPA peaks disappeared in the CPC (1/2) after 10 d of PLS immersion (Fig. 2). The residual TTCP and DCPA peaks were still present in the CPC (1/3) even when the specimens were immersed in PLS for 10 d (Fig. 3). XRD patterns of 50 % pre-dissolved CPC specimens (prepared for dissolution rate measurements) were very similar to those of the corresponding CPC specimens that had been immersed in PLS for 10 d.

The extents of HA formation in CPC with different TTCP/DCPA molar ratios ranged from 61 % to 91 % (Table 3a). Two-way ANOVA of the conversion data with CPC group (TTCP/DCPA ratio) and immersion time as independent variables showed (Table 3b) that the HA conversion was significantly affected by both the immersion time (p < 0.001) and CPC group (p < 0.001). Also, there was a significant (p = 0.018) interaction between the two main effects. As a result, the HA formation increased significantly (p < 0.05) in all CPC specimens, as the immersion time increased (Table 3a). The 1-d CPC (1/1) had significantly (p < 0.05) higher HA formation than the other two CPC groups, and the CPC (1/3) group after 10-d immersion had significantly (p < 0.05) lower HA formation than the other CPC groups (Table 3a).

### 3.4 Dissolution Rate

#### 3.4.1 Ca/P Molar Ratio

As described under the Materials and Methods section, the dual-constant composition methods measured the dissolution rates of Ca and P independently without a prior knowledge of the Ca and P contents or Ca/P ratio of the sample. The experimentally measured Ca and P dissolution rates were used to calculate the Ca/P ratio of the sample dissolved at any time period. These experimentally determined ratios of Ca/P dissolution rates (Table 4a) agree well with Ca/P ratios of the samples. In contrast, within each CPC group there were no significant differences in the ratio of Ca/P dissolution rates between the 1-d sample and the 50 % pre-dissolved sample.

ANOVA results (Table 4b) showed that the TTCP/DCPA ratio used when preparing the samples, which leads to a corresponding Ca/P ratio of the samples, had a significant effect on the ratio of Ca/P dissolution rates. Sample pretreatment (1-d sample or 50 % pre-dissolved samples), on the other hand, had no effects, and there were no interactions between the two main effects.

#### 3.4.2 Dissolution Rate

As described in the Materials and Method section, the independently measured Ca and P dissolution rates were used to calculate the dissolution rate in terms of μg of HA dissolved per square centimeter of sample surface area per min. Thus, in Table 3a the dissolution rates are expressed as the rate of Ca, P, and mass dissolved per cm^2^ of sample surface area per min [4]. There were no significant differences in Ca dissolution rates between any two groups (Table 3a). In contrast, there were significant differences in the P dissolution rates and mass dissolution rates. Two-way ANOVA results (Table 4b) indicated that sample pretreatment produced no effects in any of the dissolution rates, while the CPC group (TTCP/DCPA ratio) produced significant effects on P dissolution rate as well as on mass dissolution rate. There was a significant interaction between the two effects on the P dissolution rate.

## 4. Discussion

Because of its small particle size (1 μm), an increase in the amount of DCPA in the CPC mixture required a lower P/L ratio to produce a paste with good workability. As a result, CPCs with TTCP/DCPA molar ratios of 1/1, 1/2 and 1/3 were prepared with P/L ratios 4.0, 3.5 and 3.3, respectively. The setting times for the three CPC groups were not significantly different (Table 1) in spite of the different P/L ratios, suggesting that the ratio used for each CPC was a suitable choice in that it produced a paste with good workability while not adversely affecting the setting time.

In general, the porosity of set CPC specimens strongly depends on the P/L ratio used for preparing the cement paste [11]. In the present study, the porosity increased with decreasing P/L ratio as expected (Table 1). With the exception of CPC(1/1), the porosity increased with incubation time in the PLS. Because PLS is supersaturated with respect to HA but under-saturated with respect to TTCP and DCPA, the unreacted TTCP and DCPA in the sample would be expected to dissolve in the PLS during incubation. Although this could lead to precipitation of additional HA, the dissolution-reprecipitation process may still result in a net loss in mineral content. This may be the reason for the increased porosity observed with CPC(1/2) and CPC(1/3), which contained significant amounts of unreacted DCPA in the 1-d sample; the DCPA content diminished in the 5-d and 10-d samples (Figs 1 to 3). For CPC(1/3), significant amounts of residual DCPA was still present in the 10-d sample.

The mechanical strength of the set CPCs is an important factor for many clinical applications. Table 2 shows that the DTS values decreased with decreasing TTCP/DCPA molar ratios in the initial stage. Although the 1-d CPC(1/1) specimens showed the highest DTS value, the strength decreased after immersion in PLS, while the strengths of CPC (1/2) and CPC (1/3) were unaffected by PLS immersion. Because there was no change in sample porosity with PLS incubation for CPC (1/1) (Table 1), the decrease in strength has to be attributed to other reasons. One possible reason is the loss of unreacted, relatively large TTCP particles with incubation. XRD patterns showed that CPC (1/1) samples after 1-d immersion contained a significant amount of unreacted TTCP (Fig. 1), but the residual TTCP peak became small after immersion in PLS for 5 d and 10 d. Since there was an increase in HA content with incubation (Table 3), some of the lost TTCP appeared to be converted to HA during the incubation. While this process did not lead to an increase in sample porosity (Table 1), it may be responsible for the observed decrease in strength. This observation, however, is in contrast to that of a previous study, which showed that the DTS value of set CPC increased with immersion in simulated blood plasma [12] due to dense HA formation on the exterior surface.

The dissolution study results indicated that the TTCP/DCPA ratios used in preparing the CPC specimens had a direct effect on the ratio of Ca/P dissolution rate (Table 4), as would be expected. No significant differences were found in the Ca/P ratio between the 1-d sample and 50 %-predissolved samples in the same group, suggesting that a CPC sample would dissolve at a relatively constant rate throughout the dissolution process. The dissolution increased with decreasing TTCP/DCPA ratio, suggesting that a faster resorbable CPC material can be formulated with a lower TTCP/DCPA ratio.

## 5. Conclusion

Cements can be formulated with TTCP/DCPA molar ratios varying from 1/1 to 1/3 without affecting the setting time. Cement DTS decreased from 11.7MPa to 7.2 MPa with decreasing TTCP/DCPA molar ratios from 1/1 to 1/3. Immersion in PLS led to a decreased DTS only for CPC with TTCP/DCPA ratio of 1/1. Dissolution increased with decreasing TTCP/DCPA ratios, probably attributable to an increasing amount of Ca-deficient apatite formation.

## Figures and Tables

**Table 1a:** Setting time (ST) and porosity of CPC groups with different TTCP/DCPA molar ratios

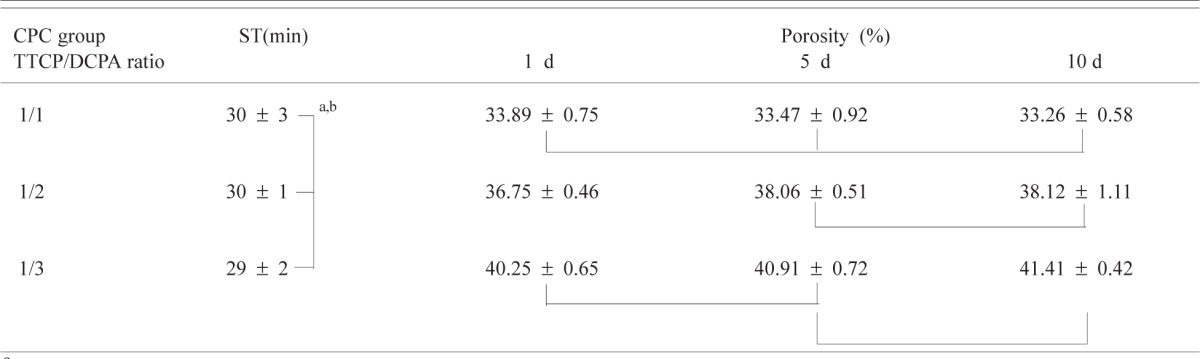

a Mean ± standard deviation (n = 3 for ST and n = 5 for porosity)

b Groups connected by a vertical or horizontal line are not significantly different (p > 0.05)

**Table 1b:** Two-way ANOVA results, main effects and interactions on porosity

Source	Significance, *p* value
Days of immersion	0.050
CPC group	< 0.001
Interaction	0.014

**Table 2a:** Diametral tensile strengths of CPC groups with different TTCP/DCPA molar ratios as a function of immersion time in PLS

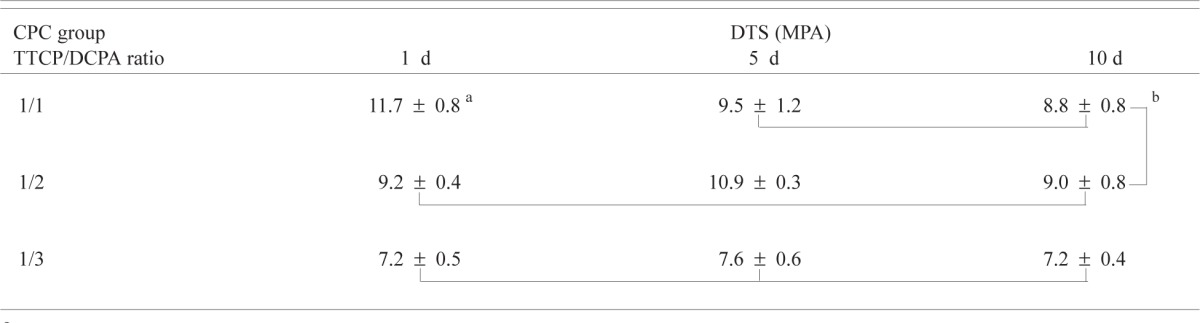

a Mean ± standard deviation (n = 5)

b Groups connected by a vertical or horizontal line are not significantly different (p > 0.05)

**Table 2b:** Two-way ANOVA results, main effects and interactions on DTS

Source	Significance, *p* value
Days of immersion	< 0.001
CPC group	< 0.001
Interaction	< 0.001

**Table 3a:** HA conversions of CPC groups with different TTCP/DCPA molar ratios as a function of immersion time in PLS

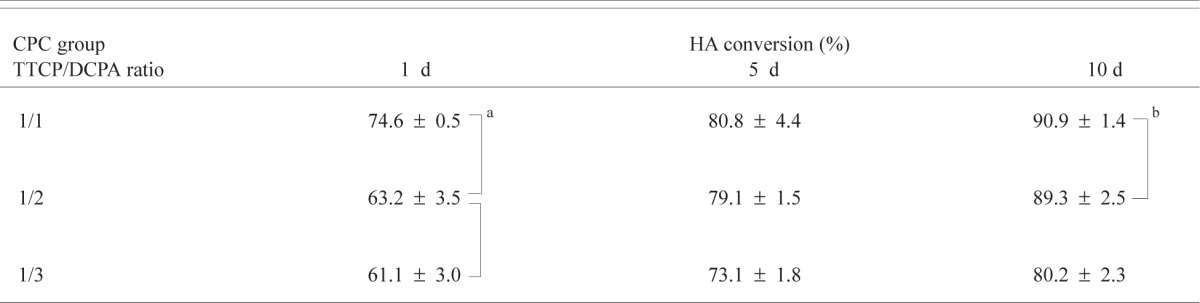

a Mean ± standard deviation (n = 3)

b Groups connected by a vertical or horizontal line are not significantly different (p > 0.05)

**Table 3b:** Two-way ANOVA results, main effects and interactions on HA conversion

Source	Significance, *p* value
Days of immersion	< 0.001
CPC group	< 0.001
Interaction	0.018

**Table 4a:** Mean dissolution rates and Ca/P ratios of various calcium phosphate samples

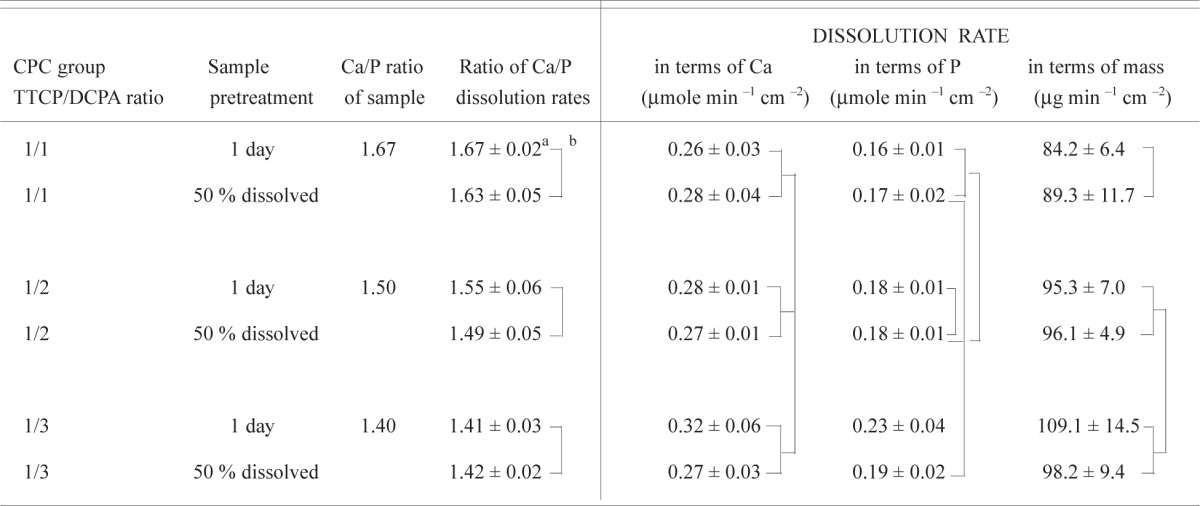

a Mean standard deviation

b Values connected by a vertical line are not significantly different

**Table 4b:** Two-way ANOVA results, main effects and interactions

Source	Ca/P Ratio	Significance, *p* value		Mass Dissolution rate
		Ca dissolution rate	P dissolution rate	
Samples pretreatment	0.062	0.212	0.303	0.637
TTCP/DCPA ratio	<0.01	0.249	<0.001	0.003
Interaction	0.130	0.056	0.025	0.179

## Footnotes

1 Certain commercial materials and equipment are identified in this paper to specify the experimental procedure. In no instance does such identification imply recommendation or endorsement by the National Institutes of Health or American Dental Association Foundation or National Institute of Standards and Technology or Nihon University or that the material or equipment identified is necessarily the best available for the purpose.
